# The Effect of Sound Lure Frequency and Habitat Type on Male *Aedes albopictus* (Diptera: Culicidae) Capture Rates With the Male *Aedes* Sound Trap

**DOI:** 10.1093/jme/tjaa242

**Published:** 2020-11-12

**Authors:** Tom Swan, Tanya L Russell, Thomas R Burkot, Jianyi Liu, Scott A Ritchie, Kyran M Staunton

**Affiliations:** 1 College of Public Health, Medical and Veterinary Sciences, James Cook University, Cairns, Australia; 2 Australian Institute of Tropical Health and Medicine, James Cook University, Cairns, Australia; 3 Verily Life Sciences, 259 East Grand Avenue, South San Francisco, CA

**Keywords:** *Aedes albopictus*, sound lure, male, wing beat frequency, mosquito surveillance

## Abstract

The global distribution of *Aedes albopictus* (Skuse) is rapidly expanding which has contributed to the emergence and re-emergence of dengue and chikungunya outbreaks. Improvements in vector surveillance are necessary to facilitate optimized, evidence-based vector control operations. Current trapping technology used to target *Ae. albopictus* and other *Aedes* species for vector surveillance are limited in both scale and scope, thus novel tools are required. Here, we evaluated the Male *Aedes* Sound Trap (MAST) for its capacity to sample male *Ae. albopictus*. Aims of this study were twofold: 1) to determine the most effective frequency for capturing male *Ae. albopictus* and 2) to investigate fine-scale variations in male *Ae. albopictus* abundance. MASTs which produced sound lure frequencies between 500 and 650 Hz captured significantly more male *Ae. albopictus* than those with sound lure frequencies set to 450 Hz. Further, the higher sound lure frequency of 700 Hz significantly reduced catches relative to 650 Hz. MASTs placed in woodland habitats captured significantly more male *Ae. albopictus* than MASTs placed near houses. These results provide baseline information for optimizing sound lure frequencies and placement of the MAST to sample male *Ae. albopictus* in remote areas.

The ‘Asian tiger mosquito’, *Aedes albopictus* (Skuse), is one of the most successful invasive insect species, largely as a result of human-aided movement over the last 40 yr (Lounibos 2002, Kraemer et al. 2015). High abundance of this species aid its ability to spread arboviruses including dengue and chikungunya (Lounibos and Kramer 2016), and its role as a nuisance species (Bonizzoni et al. 2013). Within the Indo-Pacific region, *Ae. albopictus* is wide spread, but has not yet become established on the Australian mainland, despite being found in many of the outer islands of the Torres Strait. Establishment of *Ae. albopictus* on the Australian mainland may only be a matter of time (Benedict et al. 2007, Bonizzoni et al. 2013). Once established, *Ae. albopictus* could expand its range to include most of the east coast of Australia (Russell et al. 2005, Hill et al. 2014).

Novel vector sampling traps could benefit mosquito programs to operate more effectively in remote locations. In remote areas, in particular, a low-power usage trap would be an attractive option in areas with limited, unreliable or expensive electricity. The Male *Aedes* Sound Trap (MAST), is small and practical being powered by only one AA battery (Staunton et al. 2020b). The sound emitted from the MAST emulates the frequency of female *Aedes* wing beats, a technique that has been demonstrated to effectively lure male *Aedes* (Clements 1999, Johnson et al. 2018, Rohde et al. 2019). Early traps using sound lures required bulky sound generators or tape recorders and adhesives (Kanda et al. 1987, Ikeshoji and Ogawa 1988) or an audio-oscillator powered by a 12-V battery (Balestrino et al. 2016), live animals, and/or CO_2_ (Kanda et al. 1987), all of which limit the traps utility in remote settings.

While male *Ae. albopictus* do not pose a direct public health threat (as they do not blood feed), they are a fundamental part of any *Ae. albopictus* population. Male mosquitoes typically emerge prior to females, and a trap specifically targeting males could provide early detection of a species presence in a given area. Second, various mosquito release programs have utilized *Wolbachia* transinfected *Ae. albopictus* males to suppress *Ae. albopictus* populations (Mains et al. 2016, Zheng et al. 2019). A trap specifically targeting male *Ae. albopictus* could be useful for evaluating mosquito abundance in areas before and following releases.

Only two studies have tested the relative attraction of *Ae. albopictus* to specific sound lure frequencies: one study in the laboratory (Balestrino et al. 2016), the other in the field (Ikeshoji and Ogawa 1988). Balestrino et al. (2016) found no significant difference in attraction of male *Ae. albopictus* to sound lure frequencies set to 545, 600, and 649 Hz. Ikeshoji and Ogawa (1988) tested sound lure frequencies set to 400 and 900 Hz and found significantly more male *Ae. albopictus* were captured at 400 Hz (average of 5.5 male/day) than 900 Hz (average of 0.3 male/day). It is likely that 900 Hz is outside the range of attractive frequencies for male *Ae. albopictus*. Determining specific sound lure frequencies which *Ae. albopictus* are attracted to, under field settings, is an important step toward developing an optimized and field-ready sound trap.

The MAST was developed to primarily catch male *Ae. aegypti* (L.) (Staunton et al. 2020b) and the capacity of the MAST for capturing male *Ae. albopictus* has yet to be determined. Thus, the first objective of this study was to determine the efficacy of different sound lure frequencies to lure male *Ae. albopictus* to be captured in the MAST.

Within a landscape, the abundance of mosquitoes are naturally highly heterogeneous (Burkot et al. 2018, Staunton et al. 2020a). Fine-scale variations in mosquito abundance is likely driven by a combination of factors including microclimate (Murdock et al. 2017) and environmental factors (Staunton et al. 2019b, 2020a). Understanding fine-scale variations in vector abundance is essential for effectively targeting vector control operations, such as vegetation barrier sprays (Li et al. 2010, Muzari et al. 2017) and also for understanding the fine-scale risk of mosquito exposure to residents. For example, *Ae. albopictus* have been shown to preferentially rest in vegetated areas (Rey et al. 2006); with BG-Sentinel (BGS) traps placed in shaded locations capturing significantly more *Ae. albopictus*, than those placed in locations without shade (Crepeau et al. 2013). The second objective of this study was to investigate fine-scale variations affecting male *Ae. albopictus* abundance and how MAST placement influences catch.

Here, we report field experiments which advance basic understanding of both the utility of the MAST and the comparative effect of sound lure frequencies and habitat type on capture rates of male *Ae. albopictus* with the MAST.

## Methods

### Study Site

The Torres Strait lies between the northernmost mainland point of Australia (Cape York) and the southern border of Papua New Guinea. This locality contains over 100 islands, of which 18 are inhabited (Stewart 2015). *Aedes albopictus* has been recorded on 14 of 17 surveyed islands in the Torres Strait (Muzari et al. 2019). On the islands of Erub, Badu, and Masig, a series of dengue outbreaks attributed to *Ae. albopictus* occurred between 2016 and 2017 (Muzari et al. 2019). Presently, operational mosquito surveillance by Queensland Health is limited to the two most populous islands (Horn and Thursday islands) owing to the challenging logistics and associated costs to trap mosquitoes in remote islands of the Torres Strait (most outer islands are more than 800 km from the closest mainland city of Cairns).

The Torres Strait region experiences distinct ‘dry’ (May–October) and ‘wet’ (November–April) seasons. Owing to its tropical location, temperatures vary marginally throughout the year. Average minimum and maximum temperatures are 24.4°C and 30.9°C for the ‘dry’ season and 25.8°C and 32.2°C for the ‘wet’ season respectively (Bureau of Meteorology 2020). Annual rainfall is estimated to be 1,452 mm, of which the vast majority falls in the ‘wet’ season (Bureau of Meteorology 2020).

**Fig. 1.**
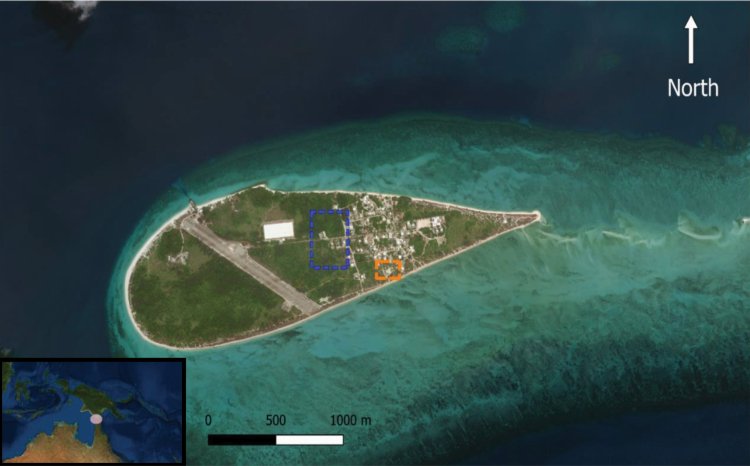
Masig Island (9.7516° S, 143.4082° E), Torres Strait, Queensland, Australia. Inset with pink circle indicates location of the Torres Strait. Orange square indicates approximate location of the frequency experiments. Blue square indicates approximate location of the habitat type experiment. Map was produced in QGIS with the World Geodetic System 1984 projection and the World Imagery (2020) layer.

Masig Island is in the central island group of the Torres Strait and is a low-elevation coral cay, 2.7 km long and 800 m at its widest point (Fig. 1). The island has a population of ca. 270 people (Australian Bureau of Statistics 2016). The major regional ecosystem on Masig Island consists of *Casuarina equisetifolia* woodland to open forest, sometimes with an understory of vine thicket species (Regional Ecosystem 3.2.6b; Neldner et al. 2019). The island has one principle village, with houses being typically bordered by a variety of native and ornamental vegetation as well as swathes of introduced weeds.

### Study Period

Three experiments were conducted on Masig Island during the wet season from 22 March 2019 to 8 April 2019 and 11–25 March 2020. There is no Bureau of Meteorology climate station on Masig Island, thus climate information were obtained from Poruma (Coconut) island, ~50 km from Masig Island. Total rainfall for the 2019 and 2020 sampling periods were 169 mm and 189 mm, respectively (Bureau of Meteorology 2020). During the 2019 sampling period, the average daily minimum and maximum temperatures were 25.6°C and 31.4°C. This was similar to the 2020 sampling period, for which the average daily minimum and maximum temperatures were 25.4°C and 31.8°C (Bureau of Meteorology 2020).

### Male *Aedes* Sound Trap

**Fig. 2.**
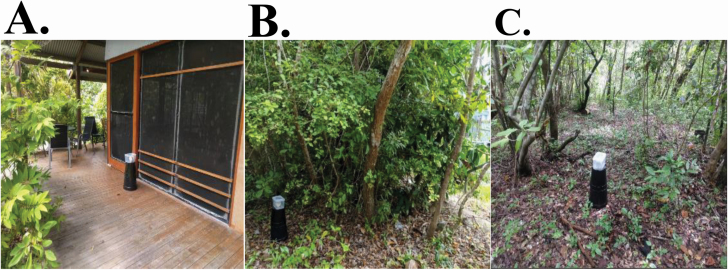
Deployment of the MAST at (A) house, (B) woodland edge, and (C) woodland habitat types on Masig Island, Torres Strait, Queensland, Australia.

The trap used in this study has been described previously (Staunton et al. 2020b). Two versions of the MAST have been developed: the MAST Sticky and the MAST Spray. The MAST Spray was used in these experiments. In brief, the MAST consists of a large black plastic base upon which sits a clear plastic rectangular container that houses captured mosquitoes. The base of the MAST was made from two inverted black buckets, one placed on top of the other. The black base serves as a visual attractant for male mosquitoes, while the sound lure (housed inside the clear container) attracted nearby male mosquitoes to fly inside the clear container (Fig. 2). Lures were programmed to produce a sinusoidal tone for 30 s on, 30 s off playback. Each lure contained a photodetector, which disabled playback between dusk and dawn. Rain guards, cut from plastic card holders (60 mm × 90 mm, Rexel, China) were placed over the lip (top of the container where the lid attaches) of each container. One day prior to the commencement of each experiment (i.e., experiment 1, 2, or 3), the inside of every clear container was sprayed once for 3 s with residual insecticide (Mortein multi-insect killer fast knockdown aerosol: 1.0 g/kg Esbiothrin 0.3 g/kg Permethrin 0.2 g/kg Imiprothrin) to prevent captured insects from escaping.

### Mosquito Sampling and Trap Processing

All traps were serviced once daily, from 9:00 a.m., with trap servicing randomized for both location and station. A ‘location’ refers to the overall area where MASTs were placed. A ‘station’ refers to the exact location where the MASTs were placed. Each MAST was deployed at least 15 m apart. GPS coordinates of stations were taken using a Garmin eTrex 10. All maps were produced using QGIS (ver. 3.6.2) with the World Geodetic System 1984 projection and the World Imagery (2020) layer. All traps were set to 70 dB sound intensity, as measured at the MAST entrance using the ‘Sound Level Meter’ Google Play application (Bolden 2020), with a Google Nexus 5x mobile phone. Captured insects were removed before examining with a Carson TV-15, TriView magnifier (15× magnification) for identifying morphologically (Zborowski 2011, Webb et al. 2016). All by-catch (non-culicines) were identified to either order or family.

### Field Trials

#### Impact of sound lure frequencies on catch rates of male *Ae. albopictus*

The aim of these experiments was to determine the comparative efficacy of different sound lure frequencies to lure male *Ae. albopictus* to be captured in the MAST.

##### Experiment 1.

**Fig. 3.**
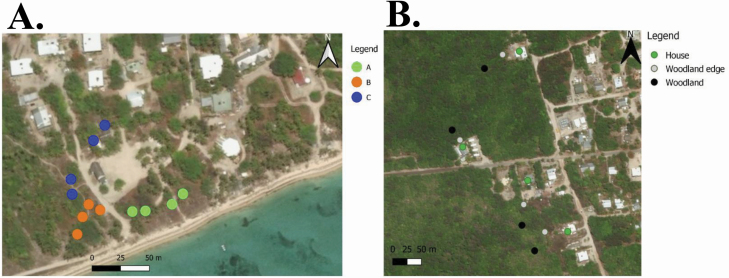
(A) stations for the three Latin square frequency experiments, (B) stations for the habitat type experiment on Masig Island, Torres Strait, Queensland, Australia. Note each station was at least 15 m apart. Map was produced in QGIS with the World Geodetic System 1984 projection and the World Imagery (2020) layer.

The range of effective sound frequencies that have previously captured male *Ae. albopictus*, being between 400 and 650 Hz, was used as initial guidance (Kanda et al. 1987, Ikeshoji and Ogawa 1988, Ikeshoji and Yap 1990, Kusakabe and Ikeshoji 1990, Balestrino et al. 2016). A 4 × 4 Latin square design was utilized and simultaneously replicated across three different locations. The three locations were all situated in costal heath, dominated by *C. equisetifolia*, *Terminalia muelleri*, and *Spinifex sericeus*. Within a location, four stations were designated, each station being at least 15 m apart. One MAST was set at each station, being a total of 12 MASTs across all three locations (Fig. 3A). In each Latin square, four MASTs playing different sound lure frequencies (450, 500, 550, and 600 Hz) were compared. The four MASTs were randomly rotated between stations of a single location daily. The entire experiment was replicated two times (*n* = 24).

##### Experiment 2.

The sound lure frequency range was expanded to 450, 600, 650, and 700 Hz to determine the relative catch rate of male *Ae. albopictus* in MASTs containing sound lures set to higher frequencies. The same Latin square design, locations, and stations as above were used (Fig. 3A). Each of the three Latin squares were replicated three times (*n* = 36). However, due to logistical constraints, bases comprised a single black bucket, instead of two black buckets stacked together, as previously deployed. The single buckets were either the top or bottom bucket of the previously stacked combination. As buckets differed in dimensions, the bases were not rotated in the Latin squares, only the clear containers with the sound lure. A previous field trial found no significant difference between catch rate of male *Ae. aegypti* between single black (bottom) buckets and two black buckets stacked together (Staunton et al. 2020, in prep).

### Influence of habitat type on catch rates of male *Ae. albopictus*

The aim of this experiment was to investigate fine-scale variations in male *Ae. albopictus* abundance and how trap placement influences catch.

#### Experiment 3.

Three defined habitat types were selected: woodland, woodland edge and house habitats (Figs 2 and 3B). Woodland and woodland edge habitats were characterized by *C. equisetifolia* woodland to open forest, with a dense sub-canopy of vine thicket species, including *Aglaia elaeagnoidea*, *Cyclophyllum* spp., *Drypetes deplanchei*, *Diospyros maritima*, *Planchonella obovate, Premna serratifolia*, and *Millettia pinnata* (Regional Ecosystem 3.2.6b; Neldner et al. 2019). Hereafter, this regional ecosystem is referred to as a woodland ecosystem. Stations in woodland edge habitats were within 5 m from the boundary of the woodland ecosystem. Stations in woodland habitats were located at least 30 m from the boundary of the woodland ecosystem. House habitats were inside the perimeter of an inhabited property. These properties were adjacent to a boundary of the woodland ecosystem. Stations in house habitats were placed within 5 m of either the front or back door. Twelve stations were selected: four in woodland, four in woodland edge, and four in house habitats. The experiment ran over a period of 7 d (*n* = 28). All traps were set to 600 Hz, based on results of experiment 1. The base of the MAST trap was made from two inverted black buckets, one placed on top of the other.

### Statistical Analyses

Graphs were produced in GraphPad Prism 8 (ver. 8.4.2), and data were analyzed in R Studio (R Core Team 2017, ver. 3.5). To investigate the impact of sound lure frequency on male *Ae. albopictus* catch rates (experiments 1 and 2) a generalized linear mixed model with a negative binomial distribution (Poisson models were consistently overdispersed) and a log-link function was performed using the *lme4* R package (ver. 1.1, Bates et al. 2015). The *aods3* R package (ver 1.1, Lesnoff and Lancelot 2018) was used to analyze overdispersion. Each frequency experiment was modeled separately. The parameter ‘frequency’ (fixed effect) was fitted to total daily catch of male *Ae. albopictus* by trap frequency, with ‘day’ and ‘station’ treated as random effects in the model. To investigate the influence of habitat type on catch rates of male *Ae. albopictus* (experiment 3), the parameter ‘habitat type’ (fixed effect) was fitted to total daily catch of male *Ae. albopictus* by habitat type with ‘day’ and ‘station’ treated as random effects in the model. For all experiments, the predictor variables were analyzed with an analysis of deviance using the *car* R package (ver. 3.0, Fox and Weisberg 2018). Finally, post-hoc tukey comparisons to determine significant differences among the estimated marginal means (least-squares means) of treatment groups were performed using the *emmeans* R package (ver. 1.4.6, Lenth et al. 2020).

## Results

### Impact of Sound Lure Frequencies on Catch Rates of Male *Ae. albopictus*

In total, 312 and 360 male *Ae. albopictus* were captured in experiments 1 and 2, respectively (Table 1). Other mosquito species captured included six male *Ae. scutellaris* (Walker), one male *Verrallina funerea* (Theobold) (Diptera: Culicidae) and individual females of *Ae. notoscriptus* (Skuse), and *Ae. albopictus* (Table 1). The most abundant other invertebrates for both experiments were ants (Formicidae) and fruit flies (Drosophilidae) (Table 1).

**Table 1.** Total taxa captured during the frequency experiments

Taxa	Experiment 1 (Hz)					Experiment 2 (Hz)				
	450	500	550	600	Total	450	600	650	700	Total
*Aedes albopictus* male	23	74	101	114	312	37	115	134	74	360
*Aedes albopictus* female	0	0	1	0	1	0	0	0	0	0
*Aedes scutellaris* male	0	0	0	0	0	0	1	2	3	6
*Aedes notoscriptus* female	0	0	0	1	1	0	0	0	0	0
*Verrallina funerea* male	1	0	0	0	1	0	0	0	0	0
Formicidae	23	23	29	20	95	45	35	62	52	194
Diptera: Drosophilidae	12	40	38	38	128	2	1	4	4	11
Orthoptera	2	1	0	1	4	2	5	2	4	13
Coleoptera	0	0	0	0	0	0	6	1	0	7
Diptera (other)	0	0	1	2	3	2	0	0	0	2
Blattodea	0	0	0	1	1	0	0	1	1	2
Lepidoptera	0	0	0	0	0	1	0	0	0	1
Araneae	1	0	0	0	1	0	0	0	0	0
Total					547					596

### Experiment 1

**Fig. 4.**
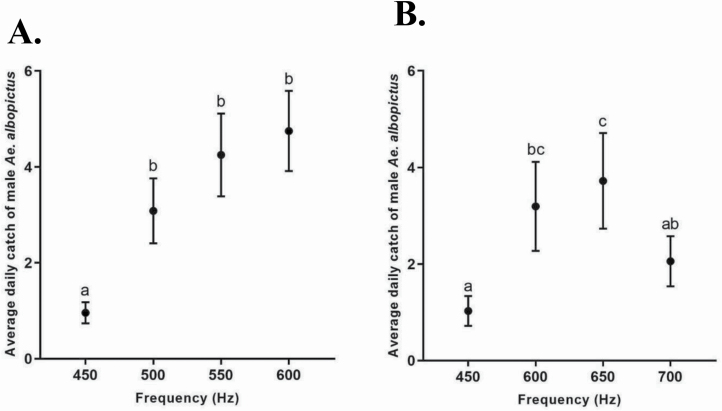
Average daily catch (± SEM) of male *Ae. albopictus* by frequency (Hz) for (A) experiment 1, and (B) experiment 2. Different letters indicate significantly different groups (*P* < 0.05, Tukey HSD, *n* = 24 for A, *n* = 36 for B).

Sound lure frequency significantly influenced the average daily catch of male *Ae. albopictus* (χ ^2^ = 39.4, df = 3, *P* < 0.0001, *n* = 24; Fig. 4A; Supp Table S1 [online only]). Traps with sound lures set to 450 Hz captured significantly less male *Ae. albopictus* (mean ± SEM; 0.96 ± 0.22) compared to traps with sound lures set to either 500 Hz (3.08 ± 0.68), 550 Hz (4.25 ± 0.86), or 600 Hz (4.75 ± 0.84). Significant differences between average daily catch of male *Ae. albopictus* were not found when sound lures were set at 500 Hz, 550 Hz and 600 Hz (Fig. 4A).

### Experiment 2

Sound lure frequency significantly influenced the average daily catch of male *Ae. albopictus* (χ ^2^ = 22.9, df = 3, *P* < 0.0001, *n* = 36; Fig. 4B; Supp Table S2 [online only]). Traps with sound lures set to 450 Hz (1.0 ± 0.31) captured significantly less male *Ae. albopictus* compared to traps with sound lures set to 600 Hz (3.2 ± 0.92) and 650 Hz (3.7 ± 0.99). Traps with sound lures set to 700 Hz (2.1 ± 0.52) captured significantly less male *Ae. albopictus* compared to traps with sound lures set to 650 Hz (Fig. 4B).

### Experiment 3

In total, 393 male *Ae. albopictus* were captured among the three habitat types (Table 2). Other mosquito species captured included: 39 male *Ae. scutellaris*, 3 female *Ae. scutellaris*, 5 male *Tripteroides magnesianus* (Edwards) (Diptera: Culicidae), and 4 female *Ae. notoscriptus* (Table 2). The most abundant other invertebrates for both experiments were ants (Formicidae) and fruit flies (Drosophilidae) (Table 2).

**Table 2.** Total taxa captured by habitat type (experiment 3)

Taxa	Woodland	Woodland edge	House	Total
*Ae. albopictus* male	188	196	9	393
*Ae. scutellaris* male	13	26	0	39
*Ae. scutellaris* female	1	2	0	3
*Ae. notoscriptus* female	3	1	0	4
*Tripteroides magnesianus* male	5	0	0	5
Formicidae	19	9	17	45
Diptera: Drosophilidae	13	7	1	21
Orthoptera	1	1	0	2
Coleoptera	0	1	1	2
Diptera: Sciaridae	0	0	1	1
Total	243	243	29	515

**Fig. 5.**
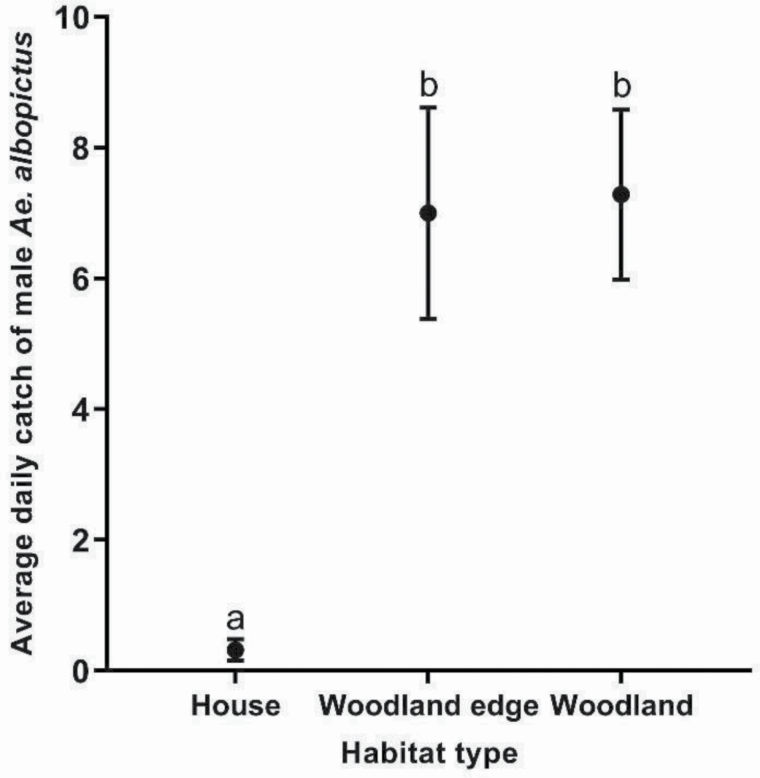
Average daily catch (± SEM) of male *Ae. albopictus* by habitat type for experiment 3. Different letters indicate significantly different groups (*P* < 0.05, Tukey HSD, *n* = 28).

Habitat type significantly influenced the average daily catch of male *Ae. albopictus* (χ ^2^ = 73.0, df = 2, *P* < 0.0001, *n* = 28; Fig. 5; Supp Table S3 [online only]). Stations at house habitats (0.32 ± 0.16) captured significantly less male *Ae. albopictus* compared to stations set at both woodland edge (7.0 ± 1.6) and woodland (7.29 ± 1.3) habitats (Fig. 5). No significant difference between average daily catch of male *Ae. albopictus* was found between woodland edge and woodland stations (Fig. 5).

## Discussion

This is the first comprehensive field study investigating catch rates of male *Ae. albopictus* relative to a range of sound lure frequencies. This work demonstrated the ability of the MAST to capture male *Ae. albopictus*. This is a promising outcome for the MAST, which owing to the sound lures’ low-power usage (the version of the sound lure tested in this study is calculated to last up to 4 mo with one AA battery) could be a useful addition for male *Ae. albopictus* surveillance in remote areas. The BGS trap is justifiably the gold standard surveillance tool for *Ae. albopictus* (Akaratovic et al. 2017, Gibson-Corrado et al. 2017), but requires power from either a 12 V battery or mains. Staunton et al. (2020b) found that the MAST caught comparable numbers of male *Ae. aegypti*, to the BGS trap. As this study was only focused on determining the efficacy of different sound lure frequencies and habitat types to capture male *Ae. albopictus*, the effectiveness of the MAST to capture male *Ae. albopictus,* relative to other mosquito traps, is unknown. Future studies concerning male *Ae. albopictus* could compare the efficacy of the MAST in capturing male mosquitoes overall, relative to well-established traps such as the BGS trap.

Low by-catch in both the present study and Staunton et al. (2020b) is supportive of the MAST being highly selective toward capturing both male *Ae. aegypti* and *Ae. albopictus*. Limited by-catch is likely a result of the MAST design. By-catch is reduced by not utilizing an olfactory lure or fan to indiscriminately attract and capture insects. Subsequently, less by-catch could reduce time involved in sorting through traps, of benefit for researchers and mosquito release programs.

The most effective sound lure frequencies for capturing male *Ae. albopictus* were between 500 and 650 Hz in these experiments on Masig Island, and this is supported by previous research. For capturing male *Ae. albopictus* under field conditions, Balestrino et al. (2016) found no significant difference in male *Ae. albopictus* catch rates for sound frequency sweeps between 500 and 650 Hz against a BGS trap with BG sentinel lure. Under laboratory conditions, Balestrino et al. (2016) found that sound frequency sweeps between 500 and 650 Hz captured significantly more male *Ae. albopictus* than fixed frequencies of 545 Hz, 600 Hz, and 649 Hz. Here, fixed frequencies were studied, where the same frequency was played for 30 s continuously. Sound frequency sweeps between 500 and 650 Hz could increase male *Ae. albopictus* catch rate with the MAST. Future studies could determine the catch rate of male *Ae. albopictus* with fixed and sound frequency sweeps of the MAST under field conditions.

Both the present study and Balestrino et al. (2016) found that frequencies above 500 Hz were effective at capturing male *Ae. albopictus*. The initial average female *Ae. albopictus* wingbeat frequency recording of 462 Hz (Ikeshoji 1981) was used as a reference for most of the earlier sound trap studies, which largely tested 400 Hz and 480 Hz for capturing male *Ae. albopictus* (Ikeshoji 1987, Kanda et al. 1987, Ikeshoji and Ogawa 1988, Ikeshoji and Yap 1990, Kusakabe and Ikeshoji 1990). Later work by Brogdon (1994) recorded mean female *Ae. albopictus* wing beat frequencies between 536 and 544 Hz. Our results support greater male *Ae. albopictus* attraction at these higher frequencies and that frequencies below 500 Hz are suboptimal for attracting male *Ae. albopictus*. The range in frequencies attractive to male *Ae. albopictus* may reflect a plausible range of female wing beat frequencies found under field conditions, encompassing different larval rearing conditions, age of females and environmental conditions (e.g., temperature), as such factors have been demonstrated to influence female wingbeat frequencies in laboratory conditions (Costello 1974, Mukundarajan et al. 2017, Villarreal et al. 2017, Staunton et al. 2019a).

Results from thixs study suggest that characteristics from the woodland and woodland edge habitat types were associated with high numbers of male *Ae. albopictus*. At least five times more male *Ae. albopictus* were captured at stations in woodland and woodland edge than at stations near houses. All traps were placed within the flight range of *Ae. albopictus* (>200m; Marini et al. 2019, Vavassori et al. 2019), which indicates that *Ae. albopictus* had a preference for inhabiting the vegetated areas within close proximity of the village. This is likely a result of these habitats being more shaded, higher in humidity and likely plentiful oviposition sites available. Previous research on *Ae. albopictus* found more eggs in oviposition traps situated in areas with vegetation and trees than in areas without vegetation and trees (Hawley 1988, Rey et al. 2006, Honório et al. 2009, Cianci et al. 2015). Shade and vegetation may thus be an important determinant of catch rate success with the MAST, as it was for both male and female *Ae. albopictus* using BGS traps (Crepeau et al. 2013). For optimizing MAST placement, future studies should investigate environmental factors important in influencing catch (e.g., amount of shade, vegetation type, and distance to households) with both the MAST and the BGS trap, as has been undertaken for optimizing BGS trap catches of *Ae. aegypti* following ‘rear and release’ programs (Staunton et al. 2019b, 2020a).

*Aedes scutellaris* was occasionally captured with MASTs in the woodland (*n* = 13), woodland edge (*n* = 26) and in one frequency experiment (*n* = 6). This is the first report of this mosquito being attracted and captured in a sound trap. In 2002, *Ae. scutellaris* was recorded as the only container-breeding *Aedes* species on Masig Island (Ritchie et al. 2002), but subsequently was likely displaced by *Ae. albopictus* (Ritchie et al. 2006). A 2016 larval survey across the Torres Strait, showed that *Ae. scutellaris* was as widespread as *Ae. albopictus,* albeit at considerably lower abundance (Muzari et al. 2019). Our result most likely reflects catch of *Ae. scutellaris* under a low abundance setting. Futures studies concerned with the use of sound to capture *Ae. scutellaris* should focus on setting MASTs in areas where this mosquito is found at higher population abundances.

Additional development of the MAST could include telemetric options, involving sensor, photographic and communication equipment, to allow traps to continuously record and upload data. This could allow traps to be ‘set-and-forget’ and would be of considerable benefit for vector surveillance in remote areas. If further suppression or mosquito control of *Ae. albopictus* was to occur in the Torres Strait, MASTs could potentially be used to remotely monitor the success of various control strategies. Additionally, MASTs could be used as a low-power surveillance tool by countries for monitoring incursions of *Ae. albopictus* and potentially other *Aedes* species.

## Conclusion

We found that sound lure frequencies at or above 500 Hz but below 700 Hz could be used for optimizing the capture of male *Ae. albopictus* on Masig Island. MASTs may therefore provide a useful tool for male *Ae. albopictus* surveillance throughout similar locations within the Torres Strait. Additionally, MASTs placed in woodland and woodland edge habitats captured at least five times more male *Ae. albopictus* than MASTs placed at house habitats. Understanding small scale variations in vector abundance is essential for effectively targeted vector control operations. Current suppression of *Ae. albopictus* on Horn and Thursday islands, by residual harborage spraying with lambda-cyhalothrin to well-shaded vegetation below 2 m in height and leaf litter at potential harborage sites (Muzari et al. 2017), could also be an appropriate tool for *Ae. albopictus* suppression on the outer islands of the Torres Strait.

## Supplementary Material

tjaa242_suppl_Supplementary_Information

## Data Availability

Data and HTML output supporting the conclusions of this article are available from the JCU Research Data repository: doi.org/10.25903/5f23bd0447ed8 (Swan 2020).
