# Supplementary material for: The Effect of Sound Lure Frequency and Habitat Type on Male Aedes albopictus (Diptera: Culicidae) Capture Rates With the Male Aedes Sound Trap
Source: J Med Entomol. 2020 Nov 12;58(2):708–16. doi: 10.1093/jme/tjaa242 (PMC7954095; doi:10.1093/jme/tjaa242)
Supplement: tjaa242_suppl_Supplementary_Information [file tjaa242_suppl_supplementary_information.docx]

## Supplementary information

Supplementary Information 1: Post-hoc tukey multiple comparison between different frequencies (experiment 1) using 95% confidence interval. Results are given on the log (not the response) scale. Adjusted P value given following tukey method for comparing a family of 4 estimates. * < 0.05, ** <0.01 , *** <0.001.

| Comparison | Estimate | S.E. | Z ratio | P value |
| --- | --- | --- | --- | --- |
| 450 - 500 Hz | -1.221 | 0.292 | -4.182 | 0.0002*** |
| 450 - 550 Hz | -1.483 | 0.284 | -5.227 | <0.0001*** |
| 450 - 600 Hz | -1.744 | 0.284 | -6.137 | <0.0001*** |
| 500 - 550 Hz | -0.262 | 0.225 | -1.168 | 0.6469 |
| 500 - 600 Hz | -0.524 | 0.221 | -2.373 | 0.0823 |
| 550 - 600 Hz | -0.261 | 0.210 | -1.243 | 0.5994 |

Supplementary Information 2: Post-hoc tukey multiple comparison between different frequencies (experiment 2) using 95% confidence interval. Results are given on the log (not the response) scale. Adjusted P value given following tukey method for comparing a family of 4 estimates. * < 0.05, ** <0.01 , *** <0.001.

| Comparison | Estimate | S.E. | Z ratio | P value |
| --- | --- | --- | --- | --- |
| 450 – 600 Hz | -1.051 | 0.307 | -3.421 | 0.0035** |
| 450 – 650 Hz | -1.438 | 0.311 | -4.621 | <0.0001*** |
| 450 – 700 Hz | -0.700 | 0.316 | -2.213 | 0.1197 |
| 600 – 650 Hz | -0.387 | 0.270 | -1.435 | 0.4775 |
| 600 – 700 Hz | 0.351 | 0.276 | 1.270 | 0.5820 |
| 650 – 700 Hz | 0.738 | 0.282 | 2.619 | 0.0437* |

Supplementary Information 3: Post-hoc tukey multiple comparison between different habitat types (experiment 3) using 95% confidence interval. Results are given on the log (not the response) scale. Adjusted P value given following tukey method for comparing a family of 3 estimates. * < 0.05, ** <0.01 , *** <0.001.

| Comparison | Estimate | S.E. | Z ratio | P value |
| --- | --- | --- | --- | --- |
| house - woodland | -3.1238 | 0.371 | -8.428 | <0.0001*** |
| house - woodland edge | -3.0260 | 0.371 | -8.164 | <0.0001*** |
| woodland – woodland edge | 0.0979 | 0.171 | 0.574 | 0.8342 |
